# Supplementary material for: Identification of photoperiod-induced specific miRNAs in the adrenal glands of Sunite sheep (Ovis aries)
Source: Front Vet Sci. 2022 Jul 22;9:888207. doi: 10.3389/fvets.2022.888207 (PMC9354845; doi:10.3389/fvets.2022.888207)
Supplement: Supplementary file 1 [file Data_Sheet_1.ZIP › Supplementary file/miRDeep2 file.zip!/miRDeep2 file/Novel_Structure/SP42A2/Novel_408.pdf]

Provisional ID	:	NC_019466.2_20465
Score total	:	2.9
Score for star read(s)	:	3.9
Score for read counts	:	-4.4
Score for mfe	:	1.9
Score for randfold	:	1.6
Score for cons. seed	:	
Total read count	:	3
Mature read count	:	1
Loop read count	:	0
Star read count	:	2

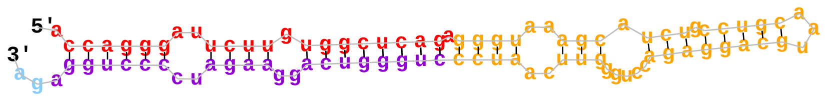

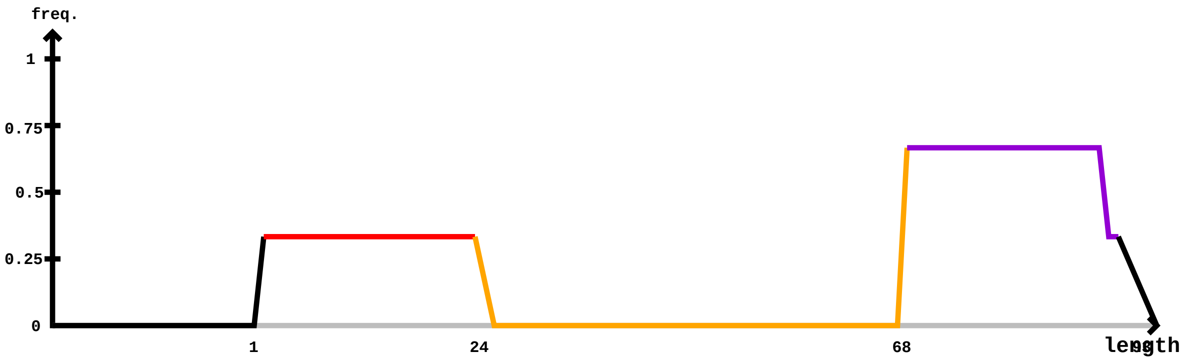


```
                      Mature                                                                   Star
5'- ucagacaguccuugggagacaccagggauucuuguggcucagaggguaaagcaucugccugcaaugcaggagaccuggguucaaucccugggucaggaagauccccuggagaa  -3'  obs
    ucagacaguccuugggagacaccagggauucuuguggcucagaggguaaagcaucugccugcaaugcaggagaccuggguucaaucccugggucaggaagauccccuggagaa        exp
    .......(((.......))).((((((..((((.((((((((.((((..(((.(((.(((((...)))))))).....)))..))))))))))))..))))..)))))).... reads  mm  sample
    ....................accagggauucuuguggcucaga..................................................................... 1      0   seq
    .......................................................................................cugggucaggaagauccccuA..... 1      1   seq
    .......................................................................................Augggucaggaagauccccugga... 1      1   seq
```
